# A Shape Memory Polymeric Shield for Protecting Corneal Endothelium During Phacoemulsification

**DOI:** 10.1167/tvst.13.4.11

**Published:** 2024-04-05

**Authors:** Yinan Liu, Yuqi Li, Jing Ji, Yubo Fan, Jing Hong, Lizhen Wang

**Affiliations:** 1 Department of Ophthalmology, Peking University Third Hospital, 49th North Garden Road, Haidian District, Beijing, China; 2 Beijing Key Laboratory of Restoration of Damaged Ocular Nerve, 49th North Garden Road, Haidian District, Beijing, China; 3 Key Laboratory of Biomechanics and Mechanobiology (Beihang University), Ministry of Education, Beijing Advanced Innovation Center for Biomedical Engineering, School of Biological Science and Medical Engineering, School of Engineering Medicine, Beihang University, Beijing, China

**Keywords:** poly-(glycerol dodecanedioate) (PGD), shape memory polymers, temperature-sensitive, phacoemulsification, corneal endothelium

## Abstract

**Background:**

The purpose of this study was to explore the protective effect of a shape memory polymeric shield on corneal endothelium during phacoemulsification in rabbits.

**Methods:**

Poly-(glycerol dodecanedioate) (PGD) with a transition temperature of 24.416°C was prepared to make a shape memory shield with a thickness of 100 µm, an arc length of 14 mm, and a radius of curvature of 8.8 mm. In the control group, a phaco-tip with bevel-down was used to simulate injury to the corneal endothelium by phacoemulsification in rabbits. In the experimental group, the pre-cooled and curled shape memory shield was injected into and removed from the anterior chamber before and after phaco-power release. Anterior segment optical coherence tomography (AS-OCT), confocal microscope, trypan blue/alizarin red staining, and scanning electron microscope were performed to measure endothelial damage after surgery.

**Results:**

One day postoperatively, the lost cell ratio of the control group and the experimental group were 28.08 ± 5.21% and 3.50 ± 1.43%, respectively (*P* < 0.0001), the damaged cell ratios were 11.83 ± 2.30% and 2.55 ± 0.52%, respectively (*P* < 0.0001), and the central corneal thicknesses (CCT) were 406.75 ± 16.74 µm and 340. 5 ±13.48 µm, respectively (*P* < 0.0001). Seven days postoperatively, the endothelial cell density (ECD) of the control group and the experimental group were 1674 ± 285/mm^2^ and 2561 ± 554/mm^2^, respectively (*P* < 0.05). The above differences were all statistically significant.

**Conclusions:**

This PGD based shape memory shield has a protective effect on corneal endothelium during phacoemulsification. It reduces postoperative corneal edema and ECD decrease in the short term after surgery.

**Translational Relevance:**

The shape memory PGD “shield” in this study may have a use in certain human patients with vulnerable corneas of low endothelial cell count or shallow anterior chambers.

## Introduction

Cataract is the leading cause of blindness worldwide.^1^ At present, phacoemulsification is the mainstream treatment, however, it may cause irreversible damage to the corneal endothelial cells with an average cell loss of approximately 5% to 18.7%.^2^^,^^3^ Human corneal endothelial cells are terminal cells and have limited regenerative potential. When the cell density falls below the threshold (approximately 400–500 cells/mm^2^) necessary for the prevention of corneal stromal swelling, corneal transparency would be lost, which may even cause corneal endothelial blindness.^4^

At present, it is generally accepted that the methods to reduce corneal endothelial damage caused by phacoemulsification include: (1) control of diabetes mellitus,^5^ (2) the “soft shell” technique using low molecular weight viscoelastic devices,^6^ and (3) reasonable setting of phaco-dynamic parameters.^7^^,^^8^ However, the effects of the above measures are relatively limited, especially for those with pre-operatively existed endothelial diseases. For these patients, it is of high clinical value to find a more effective method for corneal endothelium protection during phacoemulsification.

Material science gives us a chance to explore a material which can be easily implanted into and removed from the anterior chamber through micro incisions to protect corneal endothelium during phacoemulsification. Shape memory polymers’ (SMPs) remarkable stiffness at glass state and viscoelasticity at high elastic state make this idea possible. SMPs can be implanted to the target in a highly curled state and return to programed shapes under certain conditions. At present, more and more medical devices based on SMPs are used in clinical practices.^9^^,^^10^ However, generally speaking, most of SMPs’ transition temperatures are relatively high and difficult to be adjusted below 30°C. As a result, their mechanical properties at high elastic state are not soft enough to match the ocular tissue. To solve this problem, poly-(glycerol dodecanedioate) (PGD) is introduced in this study. Its transition temperature can be adjusted below 25°C, so that the SMPs’ properties at high elastic state are consistent with the ocular tissue.

In this study, temperature-sensitive SMPs were used to prepare a corneal endothelial shield, aiming to reduce surgical-induced damage to the corneal endothelium during phacoemulsification.

## Methods

### Preparation of PGD Material

Glycerol and dodecanedioic acid were mixed in a molar ratio of 1:1 in a nitrogen environment at 120°C for 24 hours. The synthesis product was further reacted at 120°C under a vacuum environment of −0.08 MPa for an additional 24 hours to obtain PGD prepolymer. The prepolymer was melted and poured into a polytetrafluoroethylene mold and further cured under vacuum conditions of −0.1 MPa and 120°C for 96 hours to make a shape memory shield with a thickness of 100 µm, an arc length of 14 mm, and a radius of curvature of 8.8 mm.

### Fourier Transform Infrared Spectroscopy Analysis

Arc-shaped PGD thin films prepared were placed in a 3 mL pure water environment. A Kelman phaco-tip (Alcon, USA) of the phacoemulsification platform (Centurion; Alcon, USA) was positioned with bevel-down to release power (30% phaco-power, 10 seconds phaco + 10 seconds rest, for a total of 30 cycles).

The potassium bromide (KBr) pellet method was used for Fourier transform infrared spectroscopy (FTIR) analysis of the PGD samples. First, approximately 3 µg of PGD sample before/after power release was weighed in an agate mortar. Then, approximately 200 µg of spectroscopic grade potassium bromide powder was weighed in the mortar. The sample and potassium bromide powder were thoroughly ground together, while taking care to avoid moisture. The ground mixture powder was placed in a pellet mold and pressed using a hydraulic press to form transparent sample pellets. The prepared sample pellets were subjected to FTIR analysis using an infrared spectrometer to measure the absorption peaks in the infrared range of approximately 1000 to 4000 cm^−1^ and record their spectral characteristics for analysis of the functional groups present in the sample molecules.

### Thermodynamic Properties

The thermodynamic properties of the cured PGD samples were analyzed using a DSC8000 differential scanning calorimeter (Platinum Elmer). Initially, approximately 2 mg of the PGD sample was placed in an aluminum crucible and cooled to −20°C for 1 minute to eliminate any potential thermal history effects. Next, a temperature scanning program was used, starting from −20°C and gradually increasing to 40°C, followed by a cooling phase back to −20°C, with a scanning rate of 10°C/min. The Pyris software (version 5.0; Platinum Elmer) was utilized to record the thermal flow of the PGD sample throughout the scanning process.

### Shape Fixity and Shape Recovery Testing

A bending-unbending method was used to measure the shape fixity and shape recovery capabilities of PGD material under room temperature and body temperature conditions. Specifically, rectangular PGD samples (16 × 5 × 1 mm) were immersed in a 40°C water bath for 5 minutes. After removing the sample from the water bath, it was placed into a U-shaped mold and allowed to cool and take shape at room temperature for approximately 10 minutes. The angle between the two ends of the U-shape sample was measured using a digital image analysis device and recorded as θ_max_.

The sample was then removed from the mold, and the angle between the two ends of the U-shape sample was measured, recorded as θ_fix_. The sample was immersed in a 37°C water bath, and the unfolding process was recorded in real-time using the image analysis device. The angle between the two ends of the U-shape sample at the final moment was measured and recorded as θ_i_. The shape fixity rate (Rf) and shape recovery rate (Rr) of the sample were calculated using the following formulas:

$$\begin{array}{ccc} & & R_{f}=\frac{\theta_{fix}}{\theta_{\max}}\times 100\%\\ & & R_{r}=\frac{\left(\theta_{\max}-\theta_{i}\right)}{\theta_{\max}}\times 100\% \end{array}$$

### Mechanical Performance

The mechanical properties of PGD material were determined using a universal tensile testing machine equipped with a 300N load sensor and a water bath temperature controller. First, the PGD sheet with a thickness of 1 mm was laser-cut into standard dumbbell-shaped specimens measuring 35 × 6 × 1 mm. Subsequently, the specimens (*n* = 6) were immersed in a 25°C water bath for sufficient preheating. They were then fixed onto horizontal grips of the testing machine and subjected to tensile testing at a constant temperature of 37°C in the water bath at a speed of 1 mm/min until fracture occurred. The stress-strain curves of the samples during this process were recorded and analyzed.

### Light Transmittance

The PGD material was trephined to 6-mm diameter and transferred to a 96-well plate filled with 100 uL deionized water. After balancing for 30 minutes in a 37°C incubator, the absorbance (A) of the sample in the approximately 400 to 800 nm range was detected by a full-wavelength microplate reader (Epoch2, BioTek, US). The light transmittance (T) = 10^−A^.

### CCK8 Test

The prepared PGD material was placed in 300 uL DMEM medium with 5% fetal bovine serum (FBS) and 1% penicillin-streptomycin. A phaco-tip (Kelman, Alcon, USA) with bevel-down of the Alcon Centurion platform was placed beneath the PGD material and releasing phaco-power (30% phaco-power, 10 seconds phaco + 10 seconds rest, for a total of 30 cycles). Then, the supernatant was collected. Commercialized human corneal endothelial cells (CTCC-145-HUM) were cultured in a 48-well plate. When 80% of cell confluency was reached, the culture media of the experimental group was replaced by 200 uL PGD supernatant, whereas the culture media of the control group was replaced by 200 uL fresh culture media. Then, both were incubated at 37°C for 24 hours, after which the supernatant was replaced by 500 uL fresh culture medium and 100 uL CCK8 reagent. After incubation at 37°C for 3 hours, the supernatant was transferred to a 96-well plate, and the absorbance OD value was measured at 450 nm wavelength by a microplate reader (Epoch2, BioTek, US).

The metabolic activity of the cells was expressed as “OD sample-OD blank.”

### Surgical Procedure

**Figure 1.**
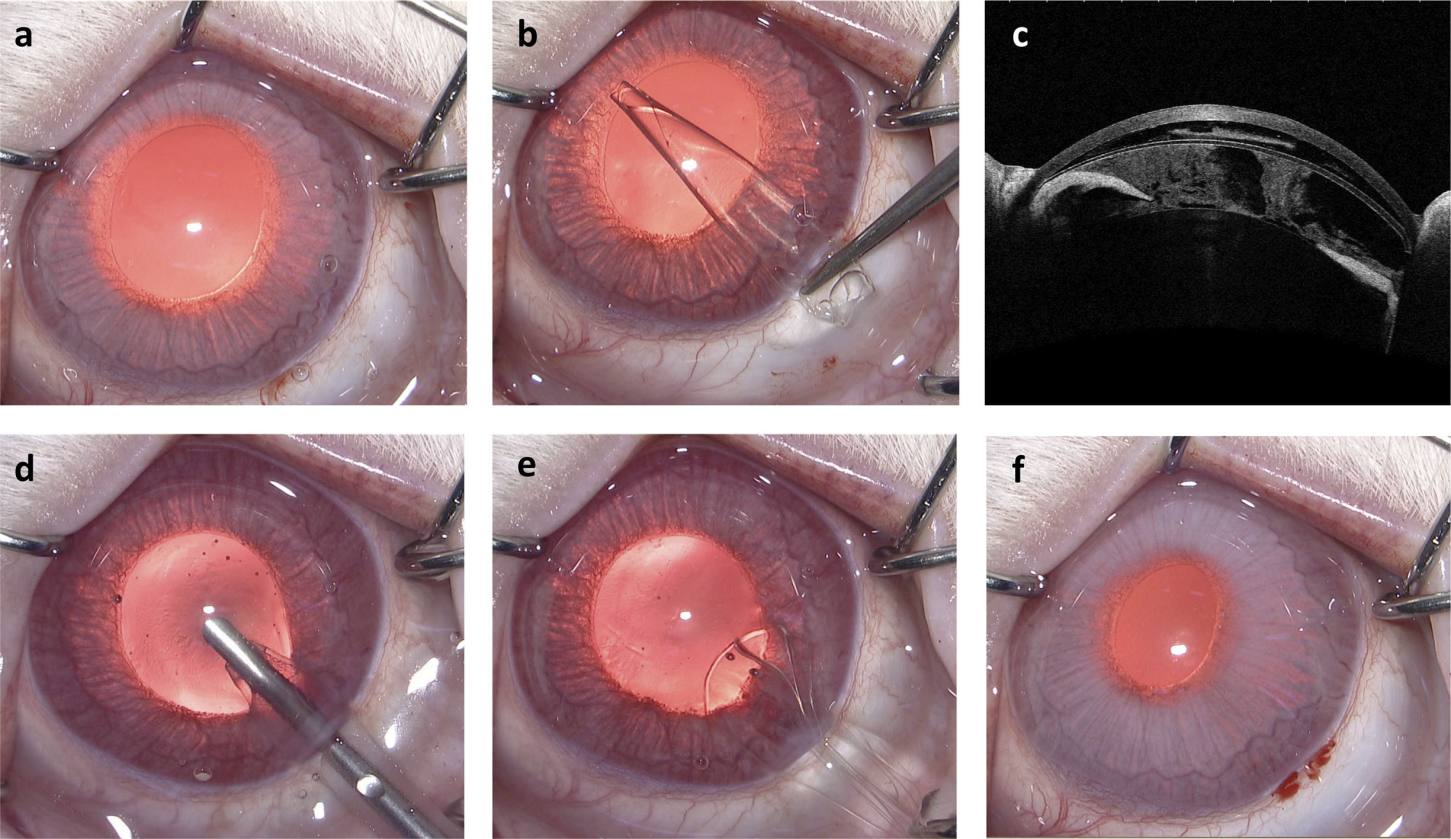
Surgical procedure of the experimental group. (**a**) A 3 mm corneoscleral incision was made and OVDs were injected into the anterior chamber; (**b**) the precooled highly curled “shape memory shield” was implanted into the anterior chamber through the incision; (**c**) the location of the “shield” in the anterior chamber shown by an AS-OCT; (**d**) a phaco-tip was located in the pupil center on the iris plane and released phaco-power; (**e**) after the injection of the OVDs, the “shield” was removed from the anterior chamber through the incision; and (**f**) the OVDs was replaced by infusion fluid and the incision closed spontaneously.

New Zealand white rabbits, male, aged 6 to 8 weeks and weighing approximately 1.5 to 2.0 kg, were purchased from Beijing Fuhao Experimental Animal Center. The study adhered to the ARVO Animal Statement and was approved by the Research Animal Ethics Committee of Peking University Third Hospital (A2023050). A total of 24 rabbits were randomly divided into 2 groups. For the experimental group, the shape memory polymeric shield was used during phacoemulsification. Whereas for the control group, we just established the phacoemulsification damage model similar to a previously published article^7^ which was summarized as follows: the surgery was performed under intramuscular injection anesthesia by 5 mg/kg tiletamine + zolazepam (Zoletil, Virbac, France) together with topical anesthesia by 0.5% proparacaine eye drops (Alcon, USA). A corneoscleral incision was made at the 10 o'clock position of the cornea with a 3.0 mm keratome blade. Functional ocular viscoelastic devices (OVDs; Alcon VisCoat, USA) were injected into the anterior chamber. A phaco-tip (Kelman, Alcon, USA) with bevel-down of the Alcon Centurion platform was placed in the pupil center on the iris plane and releasing phaco-power (30% phaco-power, 10 seconds phaco + 10 seconds rest, for a total of 30 cycles) with 40 mL/min flow, 80 cm H_2_O intraocular pressure, and 400 mm Hg vacuum. The residual OVDs were removed and replaced by infusion fluid and the corneoscleral incision was closed spontaneously at the end of the surgery. The surgical process of the control group was the same as the above-mentioned modeling process. Whereas for the experimental group, a precooled and highly curled “shape memory shield” was implanted into the anterior chamber after the injection of the OVDs, and was unfolded in the anterior chamber. After phaco-power was released, OVDs were injected into the anterior chamber again, then, the “shield” was removed through the corneoscleral incision. Intra-operative cumulative dissipated energy (CDE) was recorded. All animals were given a subconjunctival injection of 8 mg tobramycin once after the operation. The surgical procedure of the experimental group was referred to in Figure 1.

### Image Studies

Before the operation, 1 and 7 days postoperatively, the anterior segments of the rabbits were photographed with slit lamp photography (Topcon, Japan) under general anesthesia. The central corneal thickness (CCT) was measured by an anterior segment optical coherence tomography (AS-OCT; Casia2, TOMEY, Japan). The corneal endothelial cell density (ECD) was measured by a confocal microscope (Confoscan 4, Nidek, Japan) under manual mode,^11^ and the morphology of the corneal endothelial cells was observed.

### Scanning Electron Microscopy 

Scanning electron microscopy (SEM) was performed pre-operatively and 1 day and 7 days postoperatively. The rabbit eyeballs were enucleated immediately after the rabbits were humanly killed to acquire corneal samples. After phosphate-buffered saline (PBS) rinsing, the samples were immersed in 2.5% glutaraldehyde overnight at 4°C. Then, the samples were treated with 1% Osmic acid solution, ethanol solution, and the mixture of ethanol and isoamyl acetate (V/V = 1/1), as well as pure isoamyl acetate sequentially. After drying, the sample was fixed on the metal net and was sprayed with gold in a vacuum. The image was taken by a scanning electron microscope (SU8100, HITACHI, Japan).

### Trypan Blue/Alizarin Red Staining

**Figure 2.**
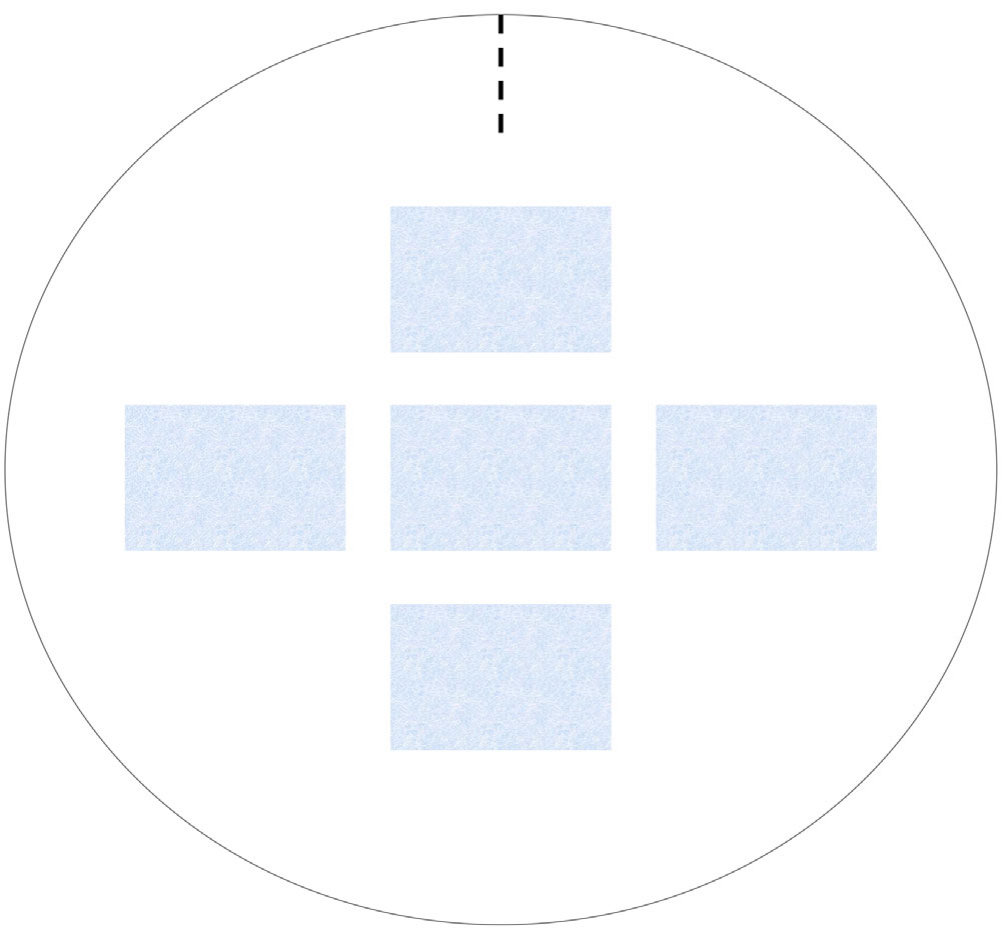
Sketch showing the five endothelial areas selected in each sample. The *dotted line* represents the radial cut at the 12 o'clock position.

Trypan blue/alizarin red staining was performed using fresh rabbit corneal samples pre-operatively and 1 day and 7 days postoperatively. The protocols were referred to in a previously published article^12^ and are briefly described as follows: the samples were treated with 0.25% trypan blue (Solarbio, China) for 90 seconds and rinsed by PBS twice. Then, they were treated with 1% alizarin red (Solarbio, China) for 60 seconds and rinsed by PBS twice. After a radial cut at the 12 o'clock position was made, the cornea was placed on a slide with endothelium upward. The staining results of the endothelium in five specific areas (at least 200 cells of each area; Fig. 2) were observed by a light microscope (Olympus, Japan). Fiji ImageJ 2.3.0 software was used to calculate the proportion of injured cells (blue staining) and lost cells (red staining of the exposed Descemet membrane) of each area and the average value was calculated as the endothelial damage level of the sample.

### Statistical Analysis

Non-parametric Wilcoxon Signed Rank Test with SPSS version 23.0 software was used to analyze the differences of ECD, CCT, damaged cell rate, and lost cell rate between the 2 groups. The difference was statistically significant when *P* < 0.05.

## Results

### Characterization of Functional Groups in PGD Before and After Surgical Treatment

**Figure 3.**
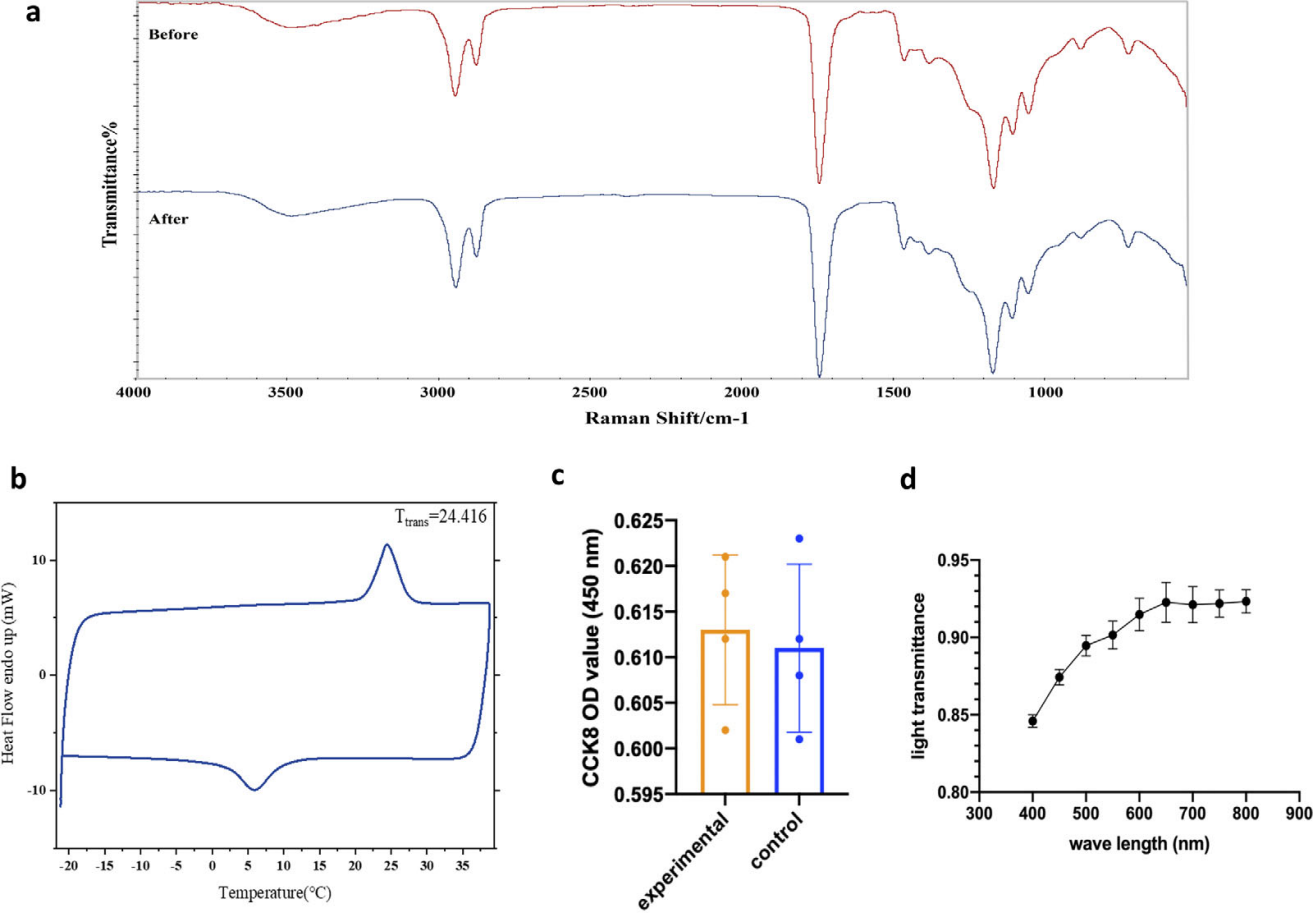
Tests of the PGD material. (**a**) Fourier transform infrared spectroscopy (FTIR) analysis. (**b**) Transition temperature. (**c**) CCK8 test. (**d**) Light transmittance.

The functional groups in PGD before and after surgical treatment were characterized, as shown in Figure 3a. There were no bond breakages or new bond formations in PGD before and after surgical treatment.

### Transition Temperature

As shown in Figure 3b, the peak temperature corresponding to the endothermic peak of PGD in the DSC heat flow curve is measured to be 24.416°C. This result indicates that during phacoemulsification, PGD can reach the transition temperature and restore its initial shape after implantation.

### Shape Memory Performance

The shape fixity rate (Rf) of PGD material was determined at 10°C, and the shape recovery rate (Rr) was measured at 25°C. The results are shown in Table 1. Both Rf and Rr were found to be higher than 98%. This indicates that PGD material can effectively fix its temporary shape at 10°C and recover its permanent shape upon releasing the temporary shape at 25°C.

**Table 1.** Shape Memory Function of PGD

Materials	Shape Fixation Rate (%)	Shape Recovery Rate (%)
PGD	98.53 ± 0.25	99.72 ± 0.26

### Mechanical Property

The mechanical properties of PGD were tested at the glassy state and high elastic state, as shown in Table 2. At the glassy state, the material exhibited a high Young's modulus and showed typical plastic mechanical behavior. At the high elastic state, PGD exhibited nonlinear elastic characteristics with a Young's modulus of 2.12 ± 0.25 MPa and a fracture strength of 1.03 ± 0.18 MPa.

**Table 2.** The Mechanical Properties of PGD at Both the Glassy and High Elastic States

State	Young's Modulus (MPa)	Tensile Strength (MPa)	Breaking Strain (%)
Rubbery state	2.12 ± 0.25	1.03 ± 0.18	62.66 ± 4.58
Glassy state	31.85 ± 3.34	2.85 ± 0.34	228.39 ± 12.72

### CCK8 Test

CCK8 test showed that the surgical degradation products of the PGD material had no significant effect on metabolic activity of the human corneal endothelial cells (Fig. 3c; *n* = 4, *P* > 0.05).

### Light Transmittance

The light transmittance of PGD material in the visible light range of approximately 400 to 800 nm was high, ranging from 84% to 93%, with a tendency to increase alongside the wavelength of the visible light (Fig. 3d; *n* = 3).

### Surgical Results

No serious complications, such as corneal endothelial decompensation occurred. After the implantation of the “shape memory shield” in the experimental group, the structure of the anterior chamber was clearly discernible, and the position of the shield was close to the corneal endothelium, leaving enough space for the operation (see Fig. 1c). In addition, because the “shield” transformed to the high elastic state and became soft in the rabbit eyes, the process of removing it through the corneoscleral incision was easy without causing additional damage to the cornea. The intra-operative CDE of the experimental group and the control group were 59.32 ± 2.13 and 57.87 ± 1.89, respectively, the difference was not significant (*P* > 0.05).

### Image Study Results

**Figure 4.**
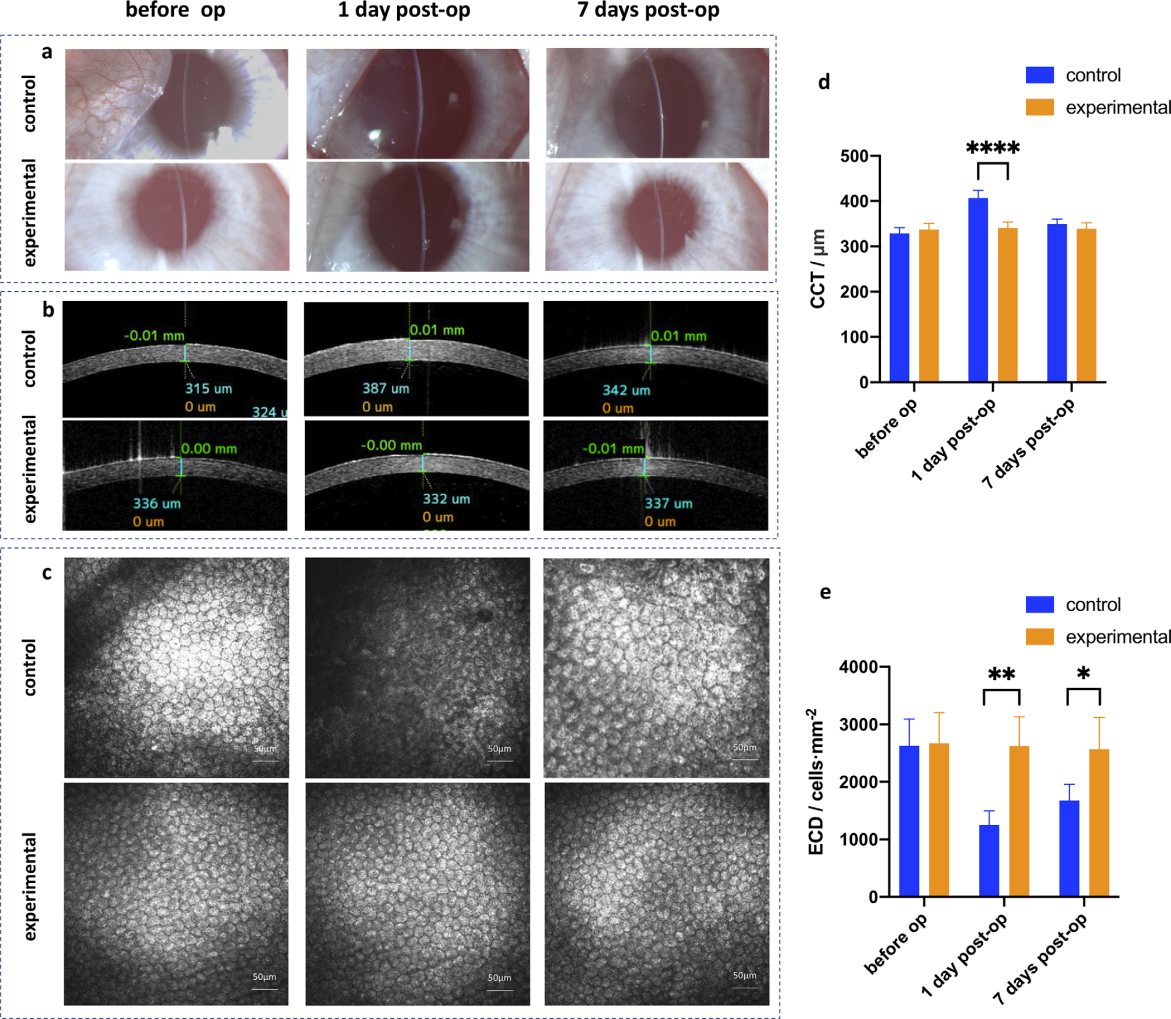
Image study results. (**a**) Anterior segment photography; (**b**) AS-OCT; (**c**) confocal microscopy; (**d**) CCT measured by AS-OCT of both groups; and (**e**) ECD measured by confocal microscopy of both groups. (*P < 0.05, **P < 0.01, ***P < 0.001, ****P < 0.0001).

Slit-lamp photography and AS-OCT both showed that there was slight corneal edema in the control group on the first day postoperatively and it disappeared on the seventh day postoperatively, however, no corneal edema was found in the experimental group both on the first and the seventh day postoperatively (Figs. 4a, 4b). Statistical analysis showed that on the first day postoperatively, the CCT of the control group and the experimental group were 406.75 ± 16.74 µm and 340.5 ± 13.48 µm, respectively, and the difference was statistically significant (Fig. 4d; *P* < 0.0001, *n* = 4). Confocal microscopy showed that on the first day postoperatively, the boundary of corneal endothelial cells (CECs) of the control group was unclear and there were large dark areas. In addition, 7 days postoperatively, the CECs of the control group enlarged in varying degrees, changing from regular hexagons to irregular polygons, whereas in the experimental group they remained unchanged (Fig. 4c). Statistical analysis showed that on the first day postoperatively, the ECD of the control group and the experimental group were 1250 ± 249/mm^2^ and 2618 ±512/mm^2^ (Fig. 4e; *P* < 0.01, *n* = 4), respectively. Seven days postoperatively, the ECD of the control group and experimental group were 1674 ± 285/ mm^2^ and 2561 ± 554/mm^2^ (see Fig. 4e; *P* < 0.05, *n* = 4), respectively, and the differences were both statistically significant.

### SEM Results

**Figure 5.**
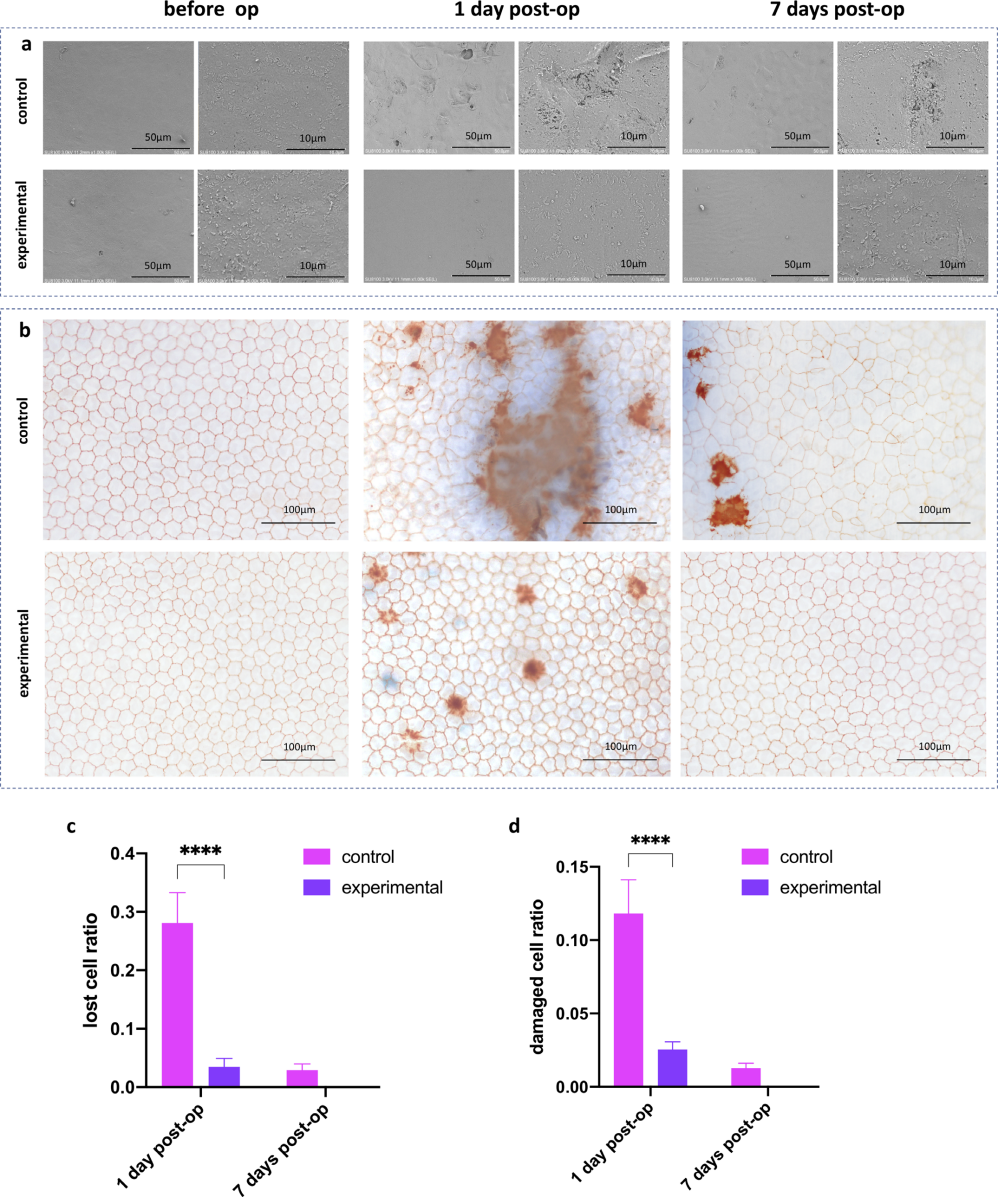
SEM (**a**) and trypan blue/alizarin red staining (**b**) results. (**c**) Cell loss ratio (red staining). (**d**) Cell damage ratio (blue staining; *P < 0.05, **P < 0.01, ***P < 0.001, ****P < 0.0001).

The CECs of the two groups were intact and regular hexagons pre-operatively. On the first and the seventh day postoperatively, the CECs’ surface of the control group occurred at different degrees of punctate hollows, and the high magnification showed that the hollows were the damages of the CECs. Whereas the morphology of CECs of the experimental group remained the same throughout the operation (Fig. 5a).

### Trypan Blue + Alizarin Red Staining Results

Figure 5b showed that the CECs of the two groups were regular hexagons pre-operatively. One day postoperatively, the CECs of the two groups showed different degrees of cell injury (blue staining) and cell loss (red staining). Besides, the CECs of the control group showed morphological changes, and some of the cells were enlarged and irregular. On the seventh day postoperatively, injury and loss of CECs of the control group was alleviated, but the cell enlargement and the irregular morphology became more obvious. However, no cell damage or cell loss was stained of the experimental group on the seventh day postoperatively, and most of the cell morphology was still regular hexagonal. Statistical analysis showed that on the first day postoperatively, the cell loss ratio of the control group and the experimental group were 28.08 ± 5.21% and 3.50 ± 1.43%, respectively (Fig. 5c; *P* < 0.0001, *n* = 4), whereas the cell damage ratios were 11.83 ± 2.30% and 2.55 ± 0.52%, respectively (Fig. 5d; *P* < 0.0001, *n* = 4), the differences were both statistically significant. When it comes to the seventh day post-operatively, the differences between groups were not statistically significant.

## Discussion

Biodegradable SMPs provide more chances for minimally invasive surgeries. Once heated, SMP devices can be folded, and programmed to fit a micro incision. Then, once cooled, they could be fixed and passed through small incisions. When heated to body temperature, the SMP devices would expand automatically again. PGD is favored in this field because of its adjustable low transition temperature and high softness at a high elastic state.

Qin et al. found that PGD materials coated with collagen and hyaluronic acid could be used as scaffolds in cartilage tissue repairing.^13^ Dai et al. fabricated PGD electrospun fibers to grow neural lineage cells.^14^ Ramaraju et al.’s research found that the PGD-SIS (small intestinal submucosa) materials can be made into implantable devices through micro incisions for various soft tissue regeneration.^15^ In our study, PGD material was used for the first time in phacoemulsification to protect CECs from surgical damage and achieved good results.

**Figure 6.**
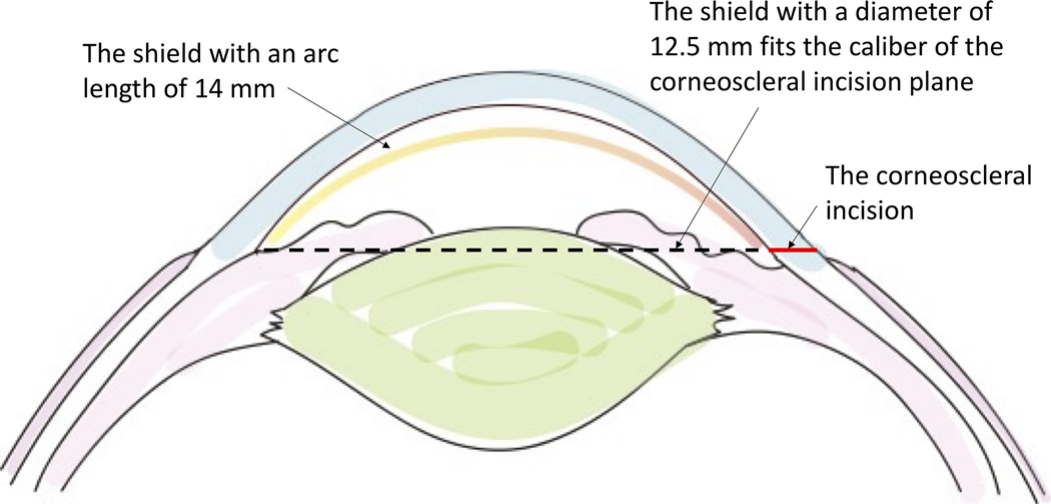
Geographic shape design of the “shape memory shield” according to the parameters of the rabbit cornea and anterior chamber.

The geographic shape design of the “shield” at the elastic state is vital for the experimental outcomes. The mean corneal radius of New Zealand white rabbits is 7.26 ± 0.26 mm; the mean horizontal and vertical diameters of the cornea are 13.41 ± 0.34 mm and 13.02 ± 0.30 mm, respectively^16^; and the peripheral corneal thickness is 407 ± 20 µm.^17^ In order to fit the above geographic shape of the rabbit cornea, we made the shape memory shield with a thickness of 100 µm, an arc length of 14 mm and a radius of curvature of 8.8 mm (Fig. 6). The curvature of the shield is slightly flatter than that of the rabbit cornea. This design is very important to prevent the shield touching the corneal endothelium in the central area. The narrow space between the corneal endothelium and the shield resulting from the mismatch in those curvatures is the key to avoid an endothelial scratch by the shield during the surgical procedure. Moreover, in order to acquire a surgical tunnel with no obstruction for the phaco-tip, the rim of the shield should be above the incision plane. In this study, we made a self-sealing corneoscleral incision to minimize the damage of the endothelial cells around the incision. In addition, the caliber of the anterior chamber on the corneoscleral incision plane is approximately 12.6 mm.^16^^,^^17^ As a result, the diameter of the shield should be no longer than 12.6 mm to fit this caliber. So, we chose 12.5 mm to be the diameter in this study to get a higher arch for more surgical space in the anterior chamber (see Fig. 6).

The mechanisms of CEC damage caused by phacoemulsification include phaco-power release, fluid turbulence, nuclear block impact, free radical attack, and intraocular pressure changes, etc.^18^ The “shape memory shield” in this study forms a physical barrier to block phaco-power release as well as fluid turbulence from the corneal endothelium. Compared with the control group, the protective effect of the “shield” on the corneal endothelium of the experimental group was obvious. First, on the first day postoperatively, the incidence of corneal edema in the experimental group was lower than that in the control group. The CCT of the control group and the experimental group was 406.75 ± 16.74 µm and 340.5 ± 13.48 µm, respectively, and the difference was statistically significant (*P* < 0.0001, *n* = 4). Second, on the seventh day postoperatively, the ECD of the control group and the experimental group were 1674 ± 285/mm^2^ and 2561 ± 554/mm^2^ (*P* < 0.05, *n* = 4) respectively. Moreover, SEM and alizarin red / trypan blue staining could reflect this protective effect more visually. On the first day postoperatively, the cell loss ratio of the control group and the experimental group were 28.08 ± 5.21% and 3.50 ± 1.43%, respectively (*P* < 0.0001, *n* = 4). In addition, the cell damage ratios were 11.83 ± 2.30% and 2.55 ± 0.52%, respectively (*P* < 0.0001, *n* = 4).

In addition to effectively protecting the corneal endothelium during phacoemulsification, the “shape memory shield” also has the following advantages: (1) the temperature-responsive characteristics enable the material to be highly folded, which is convenient for implantation into the anterior chamber through a micro incision. Moreover, the transition temperature of the “shield” is 25°C, which is lower than the temperature of the rabbit’s anterior chamber. So, when temperature falls below 10°C, the shield would be programmed into a highly curled shape to pass through the micro incision. After entering the anterior chamber, it reacts to the rabbit’s body temperature and becomes flat to complete the implantation. (2) Young's modulus of the material adapts to different stages of shield implantation to secure a successful operation. At glassy state, its Young's modulus is as high as 31.85 ± 3.34 MPa, so it is convenient to pass through the micro incision without material deformation. While in the high elastic state, its Young's modulus is as low as 2.12 ± 0.25 MPa, which matches the rabbit’s soft tissue and will not cause damage to the anterior chamber structure. Moreover, the softness of the shield at high elastic state protects the corneoscleral incision during the process of removing the shield from the anterior chamber at the end of the surgery. (3) T\the light transmittance of the material is high enough (84%-93%) at body temperature to secure a clear vision during cataract surgery. (4) Good stability with no chemical bond breakage or new bond formation during phacoemulsification to secure the safety of the surgery. (5) The CCK8 experiment showed that during the observation period, the material has no significant cytotoxicity to the human corneal endothelial cells.

This study for the first time puts forward a biomaterial strategy to protect corneal endothelium during phacoemulsification. However, one shortfall of this study is that rabbit corneal endothelial cells are capable of mitosis, so that the experimental results cannot fully represent situations in human eyes. Another shortfall is that, in this study, the corneal endothelial damage model did not include the damage of lens fragments to the corneal endothelium, but it can be predicted that the “shape memory shield” can block those damages as a physical barrier. Last but not least, the operation still requires the use of OVDs which makes the surgical process a little complicated and will prevent its routine use. Therefore, building an OVD-free surgical process by strengthening the shield's character to maintain the stability of the anterior chamber is the focus of our future study.

To sum up, the shape memory PGD “shield” in this study has a protective effect on CECs in rabbits during phacoemulsification. It acts as a physical barrier and reduces postoperative corneal edema and CEC loss in the short term after the operation. This device may have a use in certain human patients with vulnerable corneas of low endothelial cell count or shallow anterior chambers.
